# Genetic lines respond uniquely within the chicken thymic transcriptome to acute heat stress and low dose lipopolysaccharide

**DOI:** 10.1038/s41598-019-50051-0

**Published:** 2019-09-20

**Authors:** Melissa S. Monson, Angelica G. Van Goor, Michael E. Persia, Max F. Rothschild, Carl J. Schmidt, Susan J. Lamont

**Affiliations:** 1 Department of Animal Science, Iowa State University, Ames, IA USA; 2 Department of Animal and Poultry Sciences, Virginia Polytechnic Institute and State University, Blacksburg, VA USA; 3 Department of Animal and Food Sciences, University of Delaware, Newark, DE USA

**Keywords:** Transcriptomics, RNA sequencing, Thymus

## Abstract

Exposure to high temperatures is known to impair immune functions and disease resistance of poultry. Characterizing changes in the transcriptome can help identify mechanisms by which immune tissues, such as the thymus, respond to heat stress. In this study, 22-day-old chickens from two genetic lines (a relatively resistant Fayoumi line and a more susceptible broiler line) were exposed to acute heat stress (35 °C) and/or immune simulation with lipopolysaccharide (LPS; 100 µg/kg). Transcriptome responses in the thymus were identified by RNA-sequencing (RNA-seq). Expression of most genes was unaffected by heat and/or LPS in the Fayoumi line, whereas these treatments had more impact in the broiler line. Comparisons between the broiler and Fayoumi transcriptomes identified a large number of significant genes both at homeostasis and in response to treatment. Functional analyses predicted that gene expression changes impact immune responses, apoptosis, cell activation, migration, and adhesion. In broilers, acute heat stress changed thymic expression responses to LPS and could impact thymocyte survival and trafficking, and thereby contribute to the negative effects of high temperatures on immune responses. Identification of these genes and pathways provides a foundation for testing targets to improve disease resistance in heat-stressed chickens.

## Introduction

Heat-induced stress responses are well known to adversely affect poultry reproductive and metabolic performance^1–5^. Exposure to high temperatures can also suppress both innate and adaptive immunity in chickens, which facilitates secondary infections and adds to the losses attributable to heat stress^1,3–8^. As a primary immune organ, the thymus is the site of naive T-lymphocyte development and selection^9^. In broilers (meat-type chickens), chronic heat stress leads to atrophy of the thymus and other immune tissues^4–8^. Embryonic exposure to heat stress also impairs thymus development in broilers^10^. Changes in the thymus under high temperatures could impact T-cell production, and thus, lymphocyte-mediated immune capabilities.

A decline in the proportion of circulating lymphocytes compared to heterophils has been observed in heat-stressed layers (egg-type chickens) and broilers^3,7,11,12^. Increased heterophil/lymphocyte ratio also occurred in broilers during acute heat stress^13^. Within the lymphocyte compartment, both positive^14^ and negative^11,15^ effects on the percentages of peripheral CD4+ and CD8+ T-lymphocytes have been reported for high temperature exposure. Chronic heat stress decreased leukocyte protein synthesis and lymphocyte proliferation in chicken blood^12^, while another experiment using acute heat observed increased splenic lymphocyte proliferation and a shift towards more CD4+ than CD8+ T-cells^16^. Type and duration of stress are also known to modulate the response to other environmental stressors, with both inhibitory or activating effects on the immune system observed^17,18^.

High temperatures can also increase poultry susceptibility to bacterial infections. Experiments in broilers infected with *Salmonella enteritidis* demonstrated that heat stress promoted bacterial colonization of the intestinal tract, uptake into the host, and subsequent invasion of internal organs like the spleen and liver^6,19,20^. Increased intestinal permeability during heat stress also allows the gram-negative bacterial molecule lipopolysaccharide (LPS) to enter the blood stream and can generate endotoxemia, a systemic inflammatory response to LPS^21^. Immune stimulation with exogenous LPS is therefore relevant for modelling the interaction between heat and the immune system. Along with its immunostimulatory role, LPS exposure can also negatively affect the thymus by inducing thymocyte death, which results in tissue atrophy^22^.

Transcriptome-level gene expression provides a useful measure of host responses to high temperature and allows identification of adversely affected pathways and pathways that act to alleviate heat stress. Expression microarray and RNA-sequencing (RNA-seq) have been applied to many non-immune tissues to understand responses of heat-stressed chickens^23–28^. We have also used RNA-seq to profile heat and/or LPS-induced transcriptome changes in two immune tissues, the spleen^29^ and bursa of Fabricius^30^, from two chicken genetic lines at Iowa State University (ISU). The ISU Fayoumi line was imported from Egypt in 1954 to study the breed’s robustness under disease and environmental challenge, has never been selected for production traits, and has been highly (99%) inbred at ISU^31–33^. Previous work has demonstrated that the Fayoumi line is relatively resistant to a broad range of pathogens, including Marek’s disease virus, Newcastle disease virus, and *S. enteritidis*^34–36^. Conversely, the ISU broiler line is a closed population sourced in the 1990’s from an outbred broiler breeder male line and is more susceptible to heat stress and disease^23,29,35,37^. For example, the broiler line had greater changes in blood chemistry components than the Fayoumi line under the combination of heat and LPS^29^.

The objective of this study was to characterize responses to acute heat stress (35 °C for 7 hours) and/or low dose subcutaneous LPS (100 µg/kg) in the thymic transcriptome from age-matched Fayoumi and broiler chickens. We hypothesized that expression responses in the thymus will reflect the relative heat and disease resistance of each line and could impact thymocyte survival and trafficking, and thereby contribute to the negative effects of high temperatures. Understanding gene expression changes will provide insight on maintaining immune responses during heat stress and could provide targets for improving disease resistance in poultry.

## Results

### Datasets and mapping

RNA-seq datasets were generated for eight experimental groups (n = 3–4 samples/group; Table 1), which included two chicken genetic lines (Fayoumi and broiler) exposed to four treatments in a 2 × 2 design (Thermoneutral + Saline, Thermoneutral + LPS, Heat + Saline, and Heat + LPS). Sequencing depth varied greatly between individual datasets (5.6–34.4 M corrected reads; Supplementary Dataset S1), but did not differ significantly between experimental groups (p-value = 0.47) and did not affect exonic mapping percentage (p-value = 0.34). Cumulatively, 96.9% of corrected reads successfully aligned to the chicken genome and 78.9% mapped uniquely to annotated exons (Table 1). Exonic mapping percentage was also not impacted by experimental group (p-value = 0.72) or sequencing lane (p-value = 0.16). Based on these exonic read counts, 15,397 genes were sufficiently expressed (counts per million >1 in at least 3 datasets; this gives minimum of ~5 reads/gene in smallest dataset^38^) in the chicken thymus for statistical analysis, while another 7,539 genes were detected at a very low expression level.

**Table 1:** Summary of thymic RNA-seq read alignment to the chicken genome.

Group	N	Corrected Reads (M)	Mapped Reads (M)			
			Unique Exonic	Not Annotated	Not Unique	Total
Broiler Thermoneutral + Saline	3	38.1	30.4	5.3	1.3	37.0
Broiler Heat + Saline	4	58.5	45.7	7.7	3.4	56.8
Broiler Thermoneutral + LPS	4	54.6	41.8	9.4	1.9	53.1
Broiler Heat + LPS	4	52.8	40.6	8.3	2.2	51.1
Fayoumi Thermoneutral + Saline	4	75.2	60.9	10.1	1.7	72.7
Fayoumi Heat + Saline	4	75.3	59.1	12.0	1.9	72.9
Fayoumi Thermoneutral + LPS	3	66.1	53.9	8.9	1.4	64.1
Fayoumi Heat + LPS	4	76.0	59.6	12.3	1.8	73.7
Total	30	496.6	391.9	73.9	15.6	481.4
% of Corrected Reads	—	—	78.9%	14.9%	3.1%	96.9%

Lipopolysaccharide (LPS), sample number (N), million (M).

### Principal component analysis

**Figure 1:**
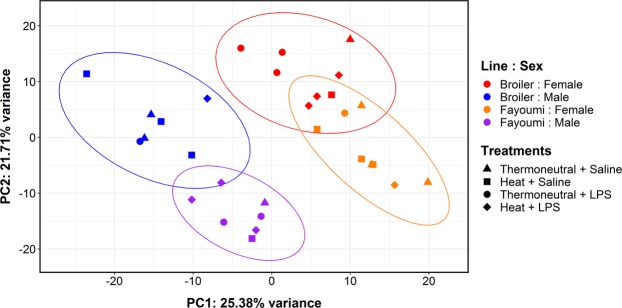
Thymic transcriptomes cluster by chicken line and sex. Principal component analysis (PCA) was performed on variance stabilized normalized read counts from the 300 most variable genes. Samples are designated by line and sex (colour) and by treatment (shape). Ellipses represent a 95% confidence interval. Lipopolysaccharide (LPS), principal component 1 (PC1), principal component 2 (PC2).

Principal component analysis (PCA) revealed that the thymic datasets clustered according to sex on PC1 (explaining 25.4% of variance) and by line on PC2 (21.7% of variance; Fig. 1). Treatment was not associated with any of the first eight PCs (cumulatively accounting for 90.7% of variance between datasets). The influence of sex on the PCA is likely attributable to the incomplete dosage compensation in chicken^39–41^, which caused sex-linked genes to be overrepresented in the 300 most variable genes used for PCA. To demonstrate this, genes on the Z and W chromosomes were excluded from PCA, which eliminated the separation by sex and made chicken genetic line the dominant effect (29.0% of variance; Supplementary Fig. S1). Thus, PCA illustrated that bird genetic background was a key determinant of thymic expression patterns and that sex needed to be included in the model in subsequent analyses (which included both autosomal and sex-linked genes).

### Differential expression

Differential expression (DE) analysis tested the main effect of experimental group (line + treatment), while also accounting for effects of sex and flowcell lane. As experimental replicate was intentionally confounded with sequencing lane, the inclusion of lane in the model removed any batch effects due to either experimental replicate or lane. Pairwise comparisons of each treatment against the Thermoneutral + Saline treatment (within line) identified 267 genes with significant DE (q-value < 0.05, |log_2_ fold change (log_2_FC)| ≥ 1.0; Supplementary Dataset S2). Consistent with PCA, comparisons of the broiler line against the Fayoumi line (within treatment) detected 1,502 significant genes. To validate the DE analysis, expression of 14 genes was measured by semi-high throughput quantitative PCR (qPCR); log_2_FC between treatments in RNA-seq and qPCR had a significant positive correlation of 0.67 and 87.5% agreement in direction when |log_2_FC| ≥ 1.0 in at least one technology (Supplementary Fig. S2).

### Broiler responses to treatment

**Figure 2:**
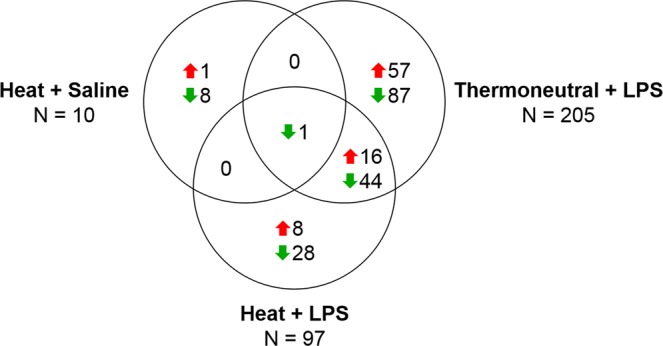
Partial overlap of significant genes revealed impacts of heat on responses to LPS in broiler thymus. Identity and direction of DE in significant genes (q-value < 0.05, |log_2_FC| ≥ 1.0) from each treatment in broiler were compared. Differential expression (DE), lipopolysaccharide (LPS), log_2_ fold change (log_2_FC), total number (N), increased (red upwards arrow), decreased (green downwards arrow).

Most expression changes in response to treatment were observed in the broiler thymus; a total of 250 genes had significant DE in at least one pairwise comparison to the Thermoneutral + Saline treatment (Supplementary Dataset S2). Only 10 genes had significant DE in response to the Heat + Saline treatment, suggesting that expression in the broiler thymus was largely unresponsive to acute heat stress (Fig. 2, Supplementary Dataset S2). Although the largest response to the Heat + Saline treatment in broiler was reduced expression of carboxylesterase 1 like 2 (*CES1L2*), most (6) of the significant changes were slight decreases in expression of genes located on the mitochondrial genome. These mitochondrial genes account for the overrepresented GO terms (“mitochondrial electron transport, cytochrome c to oxygen” and “proton transmembrane transport”) detected by PANTHER (Supplementary Dataset S3). However, Ingenuity Pathway Analysis (IPA) downstream predictions were not possible due to the small number of significant DE genes in the Heat + Saline treatment.

**Figure 3:**
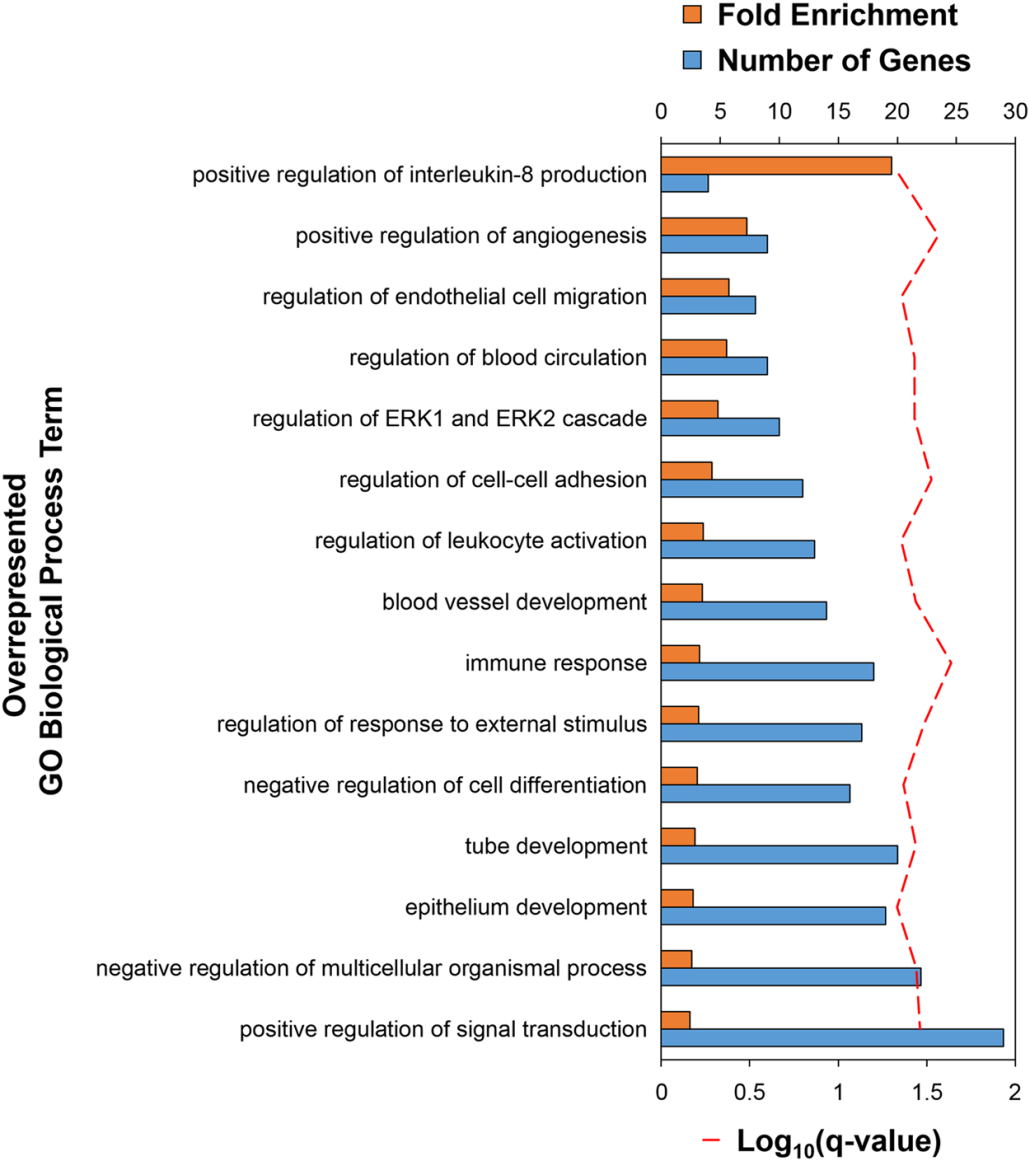
Immune response is the most significant overrepresented GO term in broiler thymus under the Thermoneutral + LPS treatment. Overrepresentation of GO Biological Process terms was tested with a Fisher’s Exact test (q-values < 0.05). The most specific overrepresented terms are shown; see Supplementary Dataset S3 for all terms. Bottom axis represents log transformed q-values (red dashed line); top axis represents both the observed number of genes (blue bars) and fold enrichment (observed number of genes/expected number of genes; orange bars). Gene Ontology (GO), lipopolysaccharide (LPS).

The Thermoneutral + LPS treatment had more impact on gene expression than heat exposure, generating significant DE in 205 genes (Fig. 2, Supplementary Dataset S2). Bactericidal permeability-increasing protein-like (*BPIL*) showed the greatest increase in expression after LPS exposure, while an uncharacterized ncRNA (*LOC112531149*) was most strongly decreased. PANTHER analysis of the significant DE genes in the Thermoneutral + LPS treatment identified overrepresentation of immune-related GO terms, including “positive regulation of interleukin-8 production”, “regulation of leukocyte activation”, and “immune response”, as well as more general terms involved in cell migration, adhesion, and differentiation, signal transduction, and development (Fig. 3, Supplementary Dataset S3). Consistent with the role of LPS as an immune stimulus, “immune response” was the most significant GO term (q-value = 0.023) and was associated with increased expression of genes such as *BPIL*, C-C motif chemokine ligand 20 (*CCL20*), fibrinogen beta chain (*FGB*), and interleukin 13 receptor subunit alpha 2 (*IL13RA2*). IPA predicted increases in “apoptosis”, “production of reactive oxygen species (ROS)”, and “cellular infiltration by phagocytes”, all consistent with a systemic inflammatory response to LPS (Table 2, Supplementary Dataset S4). Interestingly, the activation of phagocytic cells and ROS under the Thermoneutral + LPS treatment were not observed when pre-exposed to heat stress (Table 2).

**Table 2:** Top downstream functional effects of heat and/or LPS predicted for broiler thymus.

Treatment	Activated (z-score ≥ 2.0)^a^	Inhibited (z-score ≤ −2.0)^a^
Heat + Saline	—	—
Thermoneutral + LPS	• apoptosis • production of ROS • necrosis • synthesis of ROS • inhibition of cells • cellular infiltration by phagocytes • migration of smooth muscle cells • infiltration by neutrophils • cellular infiltration by myeloid cells	• vasculogenesis
Heat + LPS	• organismal death • morbidity or mortality • neonatal death • cyanosis	• quantity of cells • size of body • organization of cytoskeleton • angiogenesis • development of vasculature

^a^Functional associations (p-value < 0.05, |z-score| ≥ 2.0) were predicted using IPA (QIAGEN, Inc., https://www.qiagenbioinformatics.com/products/ingenuity-pathway-analysis). Top 10 functions (by greatest |z-score|) are shown for each comparison to the Thermoneutral + Saline treatment.

Ingenuity Pathway Analysis (IPA), lipopolysaccharide (LPS), reactive oxygen species (ROS).

Intermediate to the individual treatments, the Heat + LPS treatment induced significant DE in 97 genes (Fig. 2, Supplementary Dataset S2). Sulfotransferase family cytosolic 2B member 1-like 1 (*SULT2B1L1*) had the largest increase in expression after the Heat + LPS treatment, while *LOC112531149* remained the most decreased, as it was in the Thermoneutral + LPS treatment. “Cell adhesion” was the only GO term overrepresented within the significant genes in the Heat + LPS treatment, but this association was highly significant (q-value = 0.005; Supplementary Dataset S3). Although four genes, including fibrinogen gamma chain (*FGG*) and *FGB*, increased expression in the Heat + LPS treatment, the other “cell adhesion” associated genes, such as integrin subunit alpha 11 (*ITGA11*), were reduced. IPA further predicted that the Heat + LPS treatment could increase “morbidity or mortality”, decrease “quantity of cells” and reduce “angiogenesis”, which could have negative impacts on the broiler thymus (Table 2, Supplementary Dataset S4).

Only *CES1L2* was significantly decreased in response to all treatments, while another 60 genes were shared only between the Heat + LPS and Thermoneutral + LPS treatments (Fig. 2, Supplementary Dataset S2). These responses to LPS occurred despite the heat stress and included increased expression of *SULT2B1L1*, *FGG* and *FGB*, and decreased expression of *LOC112531149* and BMP binding endothelial regulator (*BMPER*). Another 144 genes that responded to LPS were not observed in the Heat + LPS treatment, including increases in immune genes such as *BPIL*, interleukin 1 receptor type 2 (*IL1R2*), *CCL20*, interleukin 4 induced 1 (*IL4I1*), and interleukin 18 (*IL18*). However, 36 significant genes were unique to the Heat + LPS treatment, representing DE that occurred only in the combination of these stressors; this included increased expression of neuronal pentraxin 1 (*NPTX1*) and decreased neurotrophic receptor tyrosine kinase 2 (*NTRK2*) and *ITGA11*.

### Fayoumi responses to treatment

Expression in the Fayoumi thymus was mostly unaffected by heat and/or LPS exposure, eliciting significant DE in just 21 genes in response to the Heat + LPS treatment (Table 3, Supplementary Dataset S2). The Heat + Saline and Thermoneutral + LPS treatments did not generate any significant changes in gene expression. In the Heat + LPS treatment, the greatest increases in expression were observed in the genes encoding the alpha and gamma chain precursors of fibrinogen (*FGA* and *FGG*), while chromosome 3 C1orf95 homolog (*C3H1ORF95*) had the largest decrease. Changes in *FGG*, nucleoredoxin (*NXN*), and CD34 molecule (*CD34*) were the only gene expression responses to the Heat + LPS treatment conserved between the broiler and Fayoumi lines (Supplementary Dataset S2). Due to the limited number of significant genes in the Fayoumi line, GO overrepresentation analysis and IPA were not informative, but manual analysis determined that these genes play roles in inflammation, coagulation, cell adhesion, transmembrane transport and signalling (Table 3).

**Table 3:** Differential expression of 21 genes in Fayoumi thymus in response to the Heat + LPS treatment.

Gene^a^	NCBI ID	Log_2_FC	Function(s)
*FGA*	396307	6.49	acute phase response, coagulation, cell adhesion
*FGG*	395837	2.62	acute phase response, coagulation, cell adhesion
*LOC107052991*	107052991	2.56	uncharacterized
*SLC26A4*	427845	1.64	anion transporter
*HIST1H2A4*	404299	1.38	histone, structural protein
*PLPPR2*	424477	1.19	phosphatase, signal transduction
*PCMTD1*	421114	1.08	methyltransferase, protein methylation
*MFSD2A*	419679	−1.13	phospholipid transporter
*TLL2*	107053676	−1.28	metalloproteinase, cell differentiation
*NXN*	417619	−1.33	Wnt signalling regulator, cell differentiation
*SLCO2B1*	419051	−1.36	anion transporter
*XKRX*	771005	−1.41	putative transporter
*SELP*	424364	−1.65	receptor, cell adhesion, inflammation
*MMP11*	101751203	−1.68	metalloproteinase, cell adhesion, development
*ATP10B*	416159	−1.73	phospholipid-translocating ATPase
*S1PR4*	430495	−1.91	G-protein coupled receptor
*CD34*	419856	−1.99	cell adhesion, haematopoiesis
*LOC101749178*	101749178	−2.00	uncharacterized
*VGLL3*	418462	−2.23	transcriptional cofactor, inflammation
*SPIK5*	416235	−2.57	serine protease inhibitor, T-cell differentiation
*C3H1ORF95*	421315	−3.84	uncharacterized

^a^Significant DE (q-value < 0.05, |log_2_FC| ≥ 1.0) in the Heat + LPS treatment compared to the Thermoneutral + Saline treatment.

Differential expression (DE), lipopolysaccharide (LPS), log_2_ fold change (log_2_FC).

### Comparison between chicken lines

**Figure 4:**
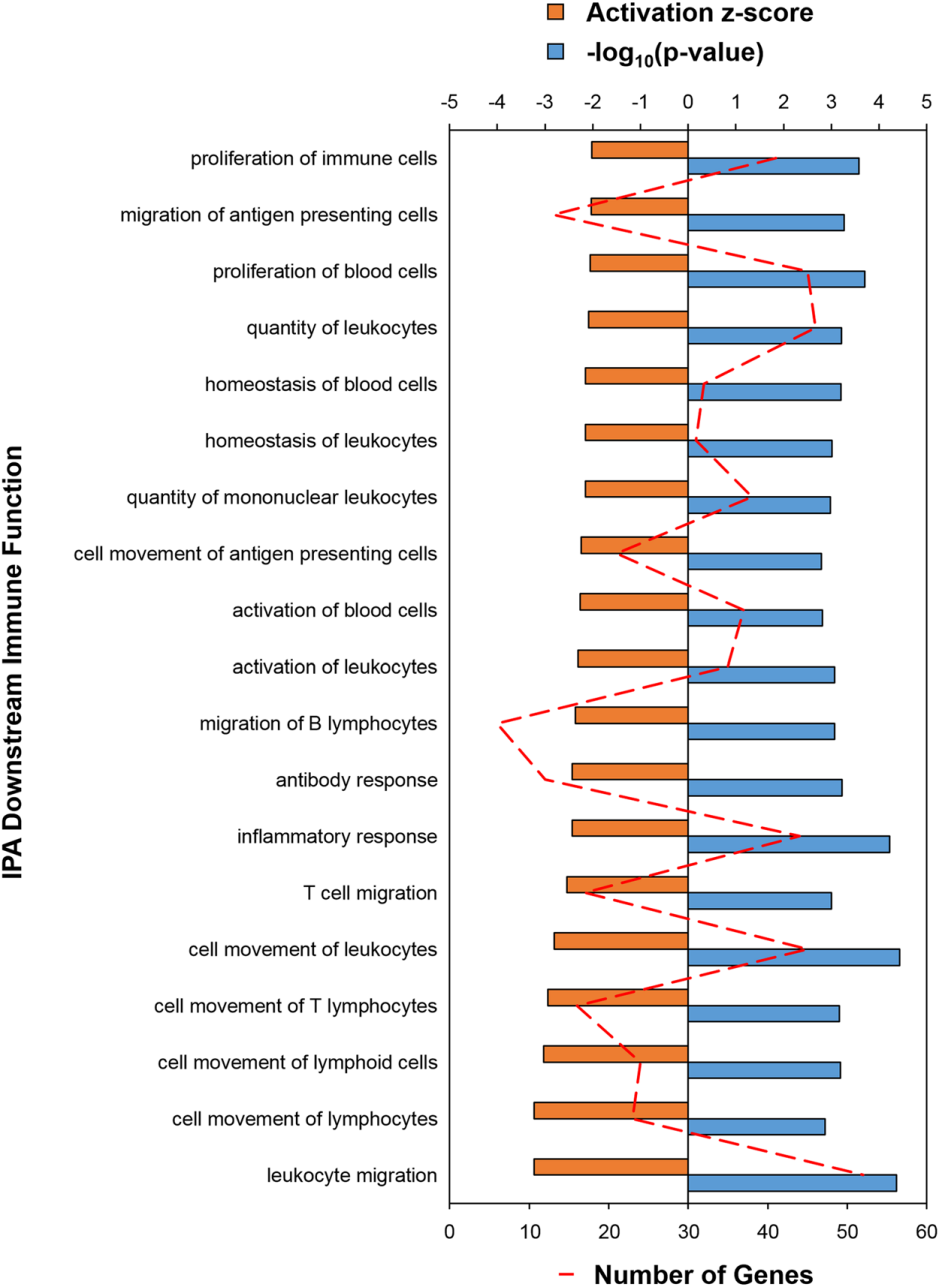
Broiler thymus predicted to have less immune activity in the Thermoneutral + Saline treatment compared to Fayoumi. IPA (QIAGEN, Inc., https://www.qiagenbioinformatics.com/products/ingenuity-pathway-analysis) was used to predict differences (p-value < 0.05; |z-score| ≥ 2.0) in broiler compared to Fayoumi. Only immune functions are shown; see Supplementary Dataset S4 for all functions. Bottom axis represents the number of genes (red dashed line); top axis represents both z-score (orange bars) and log transformed p-values (blue bars). Ingenuity Pathway Analysis (IPA).

A large number (728) of genes had significant DE in the broiler compared to the Fayoumi thymus under homeostatic conditions (Supplementary Fig. S3, Table S2). PANTHER analysis identified two overrepresented GO terms, “peptidyl-arginine ADP-ribosylation” and “immune response”, associated with the significant genes in the Thermoneutral + Saline treatment (Supplementary Dataset S3). IPA was able to detect over forty downstream functions for this comparison, including predictions of lower immune cell activation, proliferation, and migration in the broiler line compared to the Fayoumi line (Fig. 4, Supplementary Dataset S4). Over 1/3 (252) of the significant DE genes were detected only in the Thermoneutral + Saline treatment, indicating that exposure to heat or LPS eliminated some of the differences observed between lines (Supplementary Fig. S3). These included 38 genes linked by IPA to immune functions, such as *CES1L2*, heat shock protein family B member 2 (*HSPB2*), triggering receptor expressed on myeloid cells 2 (*TREM2*), and *CCL20*.

**Figure 5:**
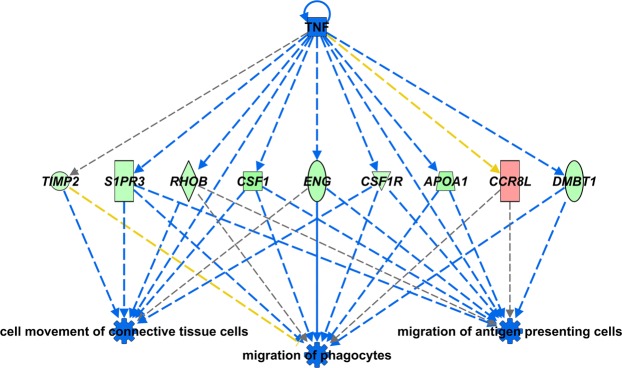
Broiler thymus predicted to have less cell migration in response to the Thermoneutral + LPS treatment compared to Fayoumi. IPA (QIAGEN, Inc., https://www.qiagenbioinformatics.com/products/ingenuity-pathway-analysis) identified a subset of genes (red = increased, green = decreased) regulated by TNF and predicted to inhibit (z-score ≤ −2.0) cell migration functions. Predicted relationships include inhibition (blue dashed line), not conclusive (grey dashed line) and inconsistent (yellow dashed line). Ingenuity Pathway Analysis (IPA), lipopolysaccharide (LPS), tumour necrosis factor (TNF).

Comparison of broiler to Fayoumi under the Heat + Saline, Thermoneutral + LPS, and Heat + LPS treatments also identified significant DE in genes not observed at homoeostasis (309, 436, and 204 genes, respectively; Supplementary Fig. S3, Dataset S2). These genes represent variable responses to heat and/or LPS in the broiler compared to the Fayoumi line. No overrepresented GO terms or significant IPA functions could be identified for the genes in the Heat + Saline treatment, although one of the largest line differences was observed in *BPIL* (higher expression in Fayoumi). Conversely, multiple gene functions were associated by IPA for the Thermoneutral + LPS treatment, including a prediction for greater “bleeding” in the broiler line, and greater “survival of organism”, “size of body” and “migration of antigen presenting cells” in the Fayoumi line (Supplementary Dataset S4). Many genes were associated with more than one downstream function, as shown for example by nine genes regulated by tumour necrosis factor (TNF) that could inhibit immune cell migration in broiler thymus (Fig. 5). For the Heat + LPS treatment, “incidence of tumor” was predicted to be higher in Fayoumi and the GO terms “cytotoxic T-cell differentiation” and “T-cell mediated immunity” were determined to be overrepresented for the genes that differed between broiler and Fayoumi. These GO terms were associated with T-cell surface glycoprotein CD8 alpha chain-like genes and solute carrier family 11 member 1 (*SLC11A1* (also known as *NRAMP1*)), which had higher expression in the broiler line (Supplementary Dataset S2).

## Discussion

The thymus is the primary immune tissue responsible for thymocyte development, including positive selection in the thymic cortex and negative selection in the medulla, and ultimately, naïve T-cell export to the periphery^9^. Heat stress can negatively impact the thymus, through both the direct effects of high temperature, and systemic signalling mediators such as corticosterone, cytokines, and acute phase response (APR) proteins. In chickens, exposure to chronic heat stress is well known to lead to thymus atrophy^4–8^, while the acute impacts of high temperature on the thymus are largely unstudied. This experiment used the thymic transcriptome to characterize acute responses to heat stress and LPS and demonstrated that chicken genetic line was a key determinant for expression changes under these treatments. This study represents the first direct comparison of the Fayoumi and broiler lines in the context of the thymus. Previous research has demonstrated significant differences between these lines in immune-related gene expression after *S. enteritidis* infection, in the immune functions of heterophils, and in splenic and bursal gene expression after heat and/or LPS exposure^29,30,35,42–44^.

Within the Fayoumi thymus, the small number of significant DE genes, observed only after exposure to the Heat + LPS treatment, suggests that this line was able to maintain homeostatic expression levels despite the environmental and immunological stressors. The Fayoumi line was derived from ancestors adapted to the high temperatures in Egypt^32,33^ and has been previously shown to have resistance to infectious diseases such as avian influenza and salmonellosis^35,45^; this is consistent with a minimal systemic impact of heat and LPS on gene expression in the thymus. Baseline DE between the lines also suggested that the Fayoumi line may have a more pre-primed immune system, based on predictions such as greater leukocyte activation and T-cell migration than the broiler. Thus, the Fayoumi thymus may sufficiently express most inflammatory regulators and other immune mediators under homeostatic conditions and, as seen here, need not change expression of these genes when responding to heat or LPS.

The largest expression changes in the Fayoumi thymus under the Heat + LPS treatment were increases in *FGG* and *FGA*, two genes that encode subunits of fibrinogen. The APR protein fibrinogen, and its thrombin-mediated cleavage product fibrin, are central to coagulation and produce cross-linked fibril chains that provide substrate for blood clot formation^46,47^. In mammals, fibrin(ogen) also serves as a platform for migrating cells, can act as a chemoattractant, and regulates inflammation by activating immune cells, especially macrophages^47–51^. Genes encoding fibrinogen subunits (*FGG* and *FGB*) were among the most significantly increased genes in the broiler after exposure to the Thermoneutral + LPS or Heat + LPS treatments, illustrating a conserved response between the two lines. *FGG* was also extremely up-regulated (log_2_FC = 28.5) by the Heat + LPS treatment in our previous experiment in the Fayoumi spleen^29^. Although the subunits of fibrinogen were not investigated, extrahepatic expression of other APR proteins has been demonstrated in chickens, including in the spleen, the bursa, and to a lesser extent, the thymus^52^. The current results suggest that systemic components of the APR may also include production of fibrinogen in the thymus. Systemic increases in fibrinogen expression could also affect the severity of heat-induced responses, as the progression from heat stress to heat stroke (severe, often fatal, organ dysfunction that impacts the nervous system) in mammals is due in part to increased and aberrant thrombosis^53,54^. Further experiments in chickens would be needed to validate the increase in fibrinogen at the protein level and to determine if this affects coagulation under heat stress.

Similar to Fayoumi, the broiler thymic transcriptome was largely resistant to the effects of acute heat, although the slight but significant decreases in mitochondrially encoded genes (including the cytochrome c oxidase subunits *COX1*, *COX2*, and *COX3*) could impact oxidative phosphorylation, and thus cellular respiration, T-cell metabolism, and activation^55,56^. In broiler, expression changes after exposure to LPS were more numerous than in either treatment involving heat stress and reflected a systemic inflammatory response and the potential for increased apoptosis. The route of LPS exposure in this study provides a systemic stimulus; therefore, many immune cells and other mediators could contribute to the gene expression changes and to the predictions of inflammation and apoptosis in the broiler thymus. Exposure to *Salmonella typhimurium*, *Escherichia coli*, and LPS have all been shown to rapidly induce transient thymic atrophy and lymphocyte depletion in the thymic cortex^22,57^. In the Themoneutral + LPS treatment, the broiler line was further predicted to have lower phagocytic cell migration than the Fayoumi line. This suggests that even though the broiler thymus is activated in response to LPS, it may not reach the levels of the Fayoumi thymus under homeostatic conditions. The relative degree of apoptosis and T-cell activation in the thymus between the two lines will need to be directly measured to test these hypotheses.

In the broiler thymus, 15 genes up-regulated by the Thermoneutral + LPS treatment have been previously shown in another broiler line to increase in the thymus in response to a longer exposure to a larger dose of LPS (50 mg/kg for 12 hours)^22^. This overlap predominately involved immune-related genes, such as fibrinogen subunits (*FGG* and *FGB*), TNFAIP3 interacting protein 3 (*TNIP3*), *IL1R2*, *CCL20*, *IL4I1*, *IL13RA2*, and *IL18*, and demonstrated their importance to thymic responses to LPS, as these genes were significant despite different lengths of exposure, dosages of LPS, bird ages and background genetics. Infection of broilers with *E. coli* via intra-airsac injection has also been shown to impact gene expression in the thymus; after 5 days of bacterial exposure, *IL1R2*, *IL13RA2* and *IL18* were all up-regulated in susceptible birds relative to non-infected or resistant birds^58^. Murine *IL18* encodes a pro-inflammatory cytokine that can, in conjunction with other cytokines, initiate thymocyte apoptosis^59^. Cell death is a normal component of thymocyte selection, but if dysregulated, can cause lymphoid depletion in the thymus^22,59^. Conversely, the receptor encoded by mammalian *IL1R2* plays an anti-inflammatory role by acting as a decoy, binds to IL1, and decreases signal transduction^60^. Best known for its role in human cancer, *IL13RA2* also encodes a decoy receptor that prevents IL13 from generating pro-apoptotic signalling through interleukin 13 receptor subunit alpha 1 (IL13RA1) and signal transducer and activator of transcription 6 (STAT6)^61,62^.

In the same broilers used in this study, *IL1R2*, *IL13RA2*, *IL18*, *BPIL*, *IL4I1*, *CCL20* and 18 other genes were also up-regulated in the spleen when exposed to LPS^29^. Fewer genes (8) shared significance between the thymus and bursa from these broilers, although the direction of DE in response to heat and/or LPS was in complete agreement with, for example, increased expression of *TNIP3*, alpha tocopherol transfer protein (*TTPA*), and *IL4I1*^30^. On the other hand, approximately 200 genes overlapped between the thymus and bursa for each of the comparisons between chicken lines (within treatment), with greater than 90% agreement on the direction of DE. This suggests that while thymic and bursal responses to treatment varied, line differences were more conserved between the two tissues.

Responses to the Heat + LPS treatment were also dependent on chicken line and tissue. Both the bursa and spleen had more significant DE than the thymus, with the greatest number of responses in the Fayoumi birds exposed to the Heat + LPS treatment^29,30^. Within the thymus, the broiler was more responsive to the Heat + LPS treatment and had potential for reduced cell adhesion, which could reduce the successful development of thymocytes and inhibit their ability to migrate to the perphery^63–65^. Addition of heat stress also prevented up-regulation of many of the immune genes that responded to the Thermoneutral + LPS treatment in broiler, including *BPIL*, *IL1R2*, *CCL20*, *IL4I1*, and *IL18*. Minimizing DE in these genes could change the thymic inflammatory response, potentially reducing over-inflammation and apoptosis or inhibiting necessary immune activation. Although further experiments would be needed to distinguish these outcomes, the addition of heat to LPS is unlikely to have a beneficial effect on the broiler thymus.

Investigating changes in the thymus transcriptome in response to acute heat stress and/or low dose LPS illuminated the distinct responses of the Fayoumi and broiler lines and provided genes and pathways that could impact thymocyte survival and trafficking under stress. The Fayoumi thymus maintained homeostatic expression levels for nearly all genes and thus was relatively resistant to heat and LPS. In the broiler thymus, acute heat also generated few significant expression changes, while LPS was predicted to initiate inflammation, immune activation, and apoptosis. Addition of heat stress supressed responses to LPS in broiler and could potentially impact cell adhesion. The predicted interactions of heat and the immune response warrant further investigation, especially *ex vivo* functional analyses, and confirmation of effects and segregating variants in commercial poultry populations. However, significantly up-regulated genes observed in multiple lines or tissues, such as *FGG*, *IL18*, *IL1R2* and *IL13RA2*, could have potential as bio-markers for stress in immune tissues and means to modulate the immune response. The significant genes and pathways from this analysis are potential targets for future efforts to improve lymphocyte-mediated immune functions in heat-stressed chickens.

## Methods

### Ethics statement

All animal experiments were approved by the Iowa State University Institutional Animal Care and Use Committee (protocol # 4-11-7128-G) and carried out according to this protocol.

### Animal experiment

This experiment utilized age-matched birds from a closed broiler line and an inbred Fayoumi line maintained at ISU. Birds were housed in floor pens and had continual *ad libitum* access to water and a corn-soy based feed formulated to meet or exceed the NRC requirements^66^. Four treatments were applied to both lines: Thermoneutral + Saline, Heat + Saline, Thermoneutral + LPS and Heat + LPS. Two experimental replicates of these treatments were performed (combined n = 26 broilers, 23 Fayoumi). Within each replicate, birds were divided at 17 days of age into four temperature controlled chambers; both Fayoumi and broilers were housed in each chamber, but lines were kept in separate pens due to body size differences. After a 5 day acclimation period at 25 °C (day 17–21), experimental temperatures were applied for 7 hours on day 22, where two chambers increased to 35 °C (heat stress) and two chambers remained at 25 °C (thermoneutral). Birds were subcutaneously injected with a commercially available LPS derived from *S. typhimurium* (L7261, Sigma-Aldrich, St. Louis, MO, USA) or phosphate buffered saline after 3.5 hours of heat exposure. A dose of 100 µg of LPS/kg of average body weight (per line on day 21) was injected (100 µL total volume) into birds in the Heat + LPS and Thermoneutral + LPS treatments; LPS dose and route were based on Kaiser *et al*., 2012, which demonstrated that subcutaneous exposure to 100 μg/kg LPS for 3 hours led to significant differential gene expression of multiple cytokines in the broiler line^37^. An equivalent volume of saline (100 µL) was given to birds in the Heat + Saline and Thermoneutral + Saline treatments. Birds were euthanized with an intravenous injection of sodium pentobarbital 3.5 hours after injections (7 hours after the start of heat exposure). Phenotypic responses to these four treatments within the Fayoumi and broiler lines (which included the individuals in this study) were evaluated in a previous publication^29^. For this study, thymus tissue samples were collected into RNAlater (Ambion, Inc., Austin, TX, USA), perfused at room temperature for 24 hours, and stored at −80 °C.

### RNA isolation and sequencing

Thymic tissue samples (n = 4 samples/treatment/line, except n = 3 for the Fayoumi Thermoneutral + LPS group, 31 samples total) were harvested from the same birds used by Monson *et al*., 2018 and Van Goor *et al*., 2017 to investigate splenic and bursal transcriptome responses to heat stress and/or LPS^29,30^. RNA was isolated from homogenized tissues using the RNAqueous Total RNA Isolation kit (Ambion, Inc.), followed by DNase treatment with the DNA-*free*™ DNA Removal kit (Ambion, Inc.). Resulting RNA samples were high quality (RNA Integrity Number (RIN) ≥8.3; average RIN = 9.4), as measured on the Eukaryote Total RNA Nano chip (2100 Bioanalyzer, Agilent Technologies, Santa Clara, CA, USA). Barcoded cDNA libraries were constructed from 0.5 µg total RNA input (TruSeq RNA Library Preparation kit v2, Illumina, Inc., San Diego, CA, USA) and confirmed as high quality using the DNA 1000 chip (2100 Bioanalyzer, Agilent Technologies). Libraries were multiplexed in a randomized block design (n = 8 samples/lane, 4 lanes total, see Supplementary Dataset S1 for lane assignments) and sequenced (100 bp single-end reads) on the HiSeq 2500 (Illumina, Inc.) at the ISU DNA Facility (Ames, IA, USA).

### RNA-seq bioinformatic analysis

Raw reads from each RNA-seq dataset were filtered and trimmed using Trimmomatic 0.32^67^ and the FastX Toolkit^68^ as previously described^30^. Quality of raw and corrected datasets was accessed using FastQC 0.10.1^69^. STAR 2.5.3a^70,71^ was used to align corrected reads to the chicken reference genome Galgal6a (GCA_000002315.5, NCBI Annotation Release 104; 24,403 genes), samtools 1.8^72^ to sort mapped reads by name, and HTSeq 0.9.1^73^ to count reads aligned to each gene as previously described^30^. One outlier (apparently a technical error in sample identity) was detected by PCA and was excluded from the analysis (leaving n = 3 in the broiler Thermoneutral + Saline group). PCA was performed in pcaExplorer 2.6.0^74,75^ using read counts from the 300 genes that contributed most to the variance in the transcriptome; these counts were normalized for library depth and variance-stabilizing transformed in DESeq2 1.20.0^76^.

### Differential expression analysis

Genes with low expression (<~5 reads/gene in the smallest dataset) were filtered out (threshold = count per million >1 in ≥3 datasets) of all datasets (n = 30 total) as recommended by Chen *et al*.^38^. DE analysis was performed using a negative binomial generalized linear model (GLM) in DESeq2 1.20.0^76^. Default parameters for DESeq2 were utilized, except independent filtering of the test results was not performed, as lowly expressed genes were removed prior to DE analysis. The factors in the GLM included experimental group (line + treatment), sex, and flowcell lane (design = ~sex + lane + group). As the factors of experimental replicate and flowcell lane were confounded (see Supplementary Dataset S1), lane also accounted for the effect of replicate. Log_2_ fold changes (log_2_FC) were calculated for each pairwise comparison between groups and Benjamini-Hochburg False Discovery Rate (FDR)-adjusted p-values (q-values) were assigned from Wald inferences tests. Genes with a q-value < 0.05 and |log_2_FC| ≥ 1.0 were considered to have significant DE. Two methods were used to investigate the functional effects of DE. A Fisher’s Exact test with multiple test correction (q-value < 0.05) was used in PANTHER 13.1^77,78^ to identify overrepresented GO Biological Process terms associated with the significant DE genes. IPA (QIAGEN, Inc., https://www.qiagenbioinformatics.com/products/ingenuity-pathway-analysis, Redwood City, CA, USA) was employed to identify downstream functions associated (p-value < 0.05) with the significant DE genes. Potential biological impacts of these functions were predicted by filtering for activation (z-score ≥ 2.0) or inhibition (z-score ≤ −2.0).

### Semi-high throughput qPCR validation

To validate the DE results from DESeq2, semi-high throughput qPCR was performed on the Biomark HD system (Fluidigm Corp., San Francisco, CA, USA) using the same procedure and primers as Monson *et al*.^30^. Briefly, cDNA was generated from 50 ng of RNA/sample (except T2010 (11 ng) and T2038 (43 ng)), pre-amplified in 12 cycles of multiplexed PCR, and expression measured in triplicate on a 192.24 Integrated Fluidic Circuit (IFC; Fluidigm Corp.) as previously described^30^. Raw threshold cycle (*C*t) values for each triplicate were filtered for melting curve specificity using Fluidigm Real-Time PCR Analysis software 4.3.1 (Fluidigm Corp.), for repeatability (at least 2 *C*t values/triplicate), and for consistency (standard deviation <2). Two genes (interleukin 10 (*IL10*) and interferon gamma (*IFNG*)) in the previous experiment^30^ were excluded from this analysis due to low counts in the thymic RNA-seq. For each of the other 14 test genes, *C*t values were normalized to hexose-6-phosphate dehydrogenase (*H6PD*) and averaged by group (at least 3 biological replicates/group). Log_2_FC in broiler and Fayoumi for each treatment compared to the Thermoneutral + Saline treatment were calculated using the delta delta *C*t (2^−ΔΔ*C*t^) method^79^. The consistency of Biomark qPCR and RNA-seq log_2_FC was evaluated in JMP Pro 12.0.1 (SAS Institute, Inc., Cary, NC, USA) through Pearson correlation.

## Supplementary information

Supplementary Information

Supplementary Dataset S1

Supplementary Dataset S2

Supplementary Dataset S3

Supplementary Dataset S4

## Data Availability

The RNA-seq datasets generated and analysed during the current study are available in the ArrayExpress database (http://www.ebi.ac.uk/arrayexpress) under accession number E-MTAB-6290. The DE results which support the conclusions of this article are provided in Table 3 and Supplementary Dataset S2. Raw Biomark qPCR data generated for validation of this study are available from the corresponding author upon reasonable request.
