# Supplementary material for: Genetic lines respond uniquely within the chicken thymic transcriptome to acute heat stress and low dose lipopolysaccharide
Source: Sci Rep. 2019 Sep 20;9:13649. doi: 10.1038/s41598-019-50051-0 (PMC6754502; doi:10.1038/s41598-019-50051-0)
Supplement: Supplementary file 1 — Supplementary Information [file 41598_2019_50051_MOESM1_ESM.docx]

**Genetic lines respond uniquely within the chicken thymic transcriptome to acute heat stress and low dose lipopolysaccharide**

Melissa S. Monson^1^†, Angelica G. Van Goor^1^†, Michael E. Persia^2^, Max F. Rothschild^1^, Carl J. Schmidt^3^ and Susan J. Lamont^1^

^1^Department of Animal Science, Iowa State University, Ames, IA, USA

^2^Department of Animal and Poultry Sciences, Virginia Polytechnic Institute and State University, Blacksburg, VA, USA

^3^Department of Animal and Food Sciences, University of Delaware, Newark, DE, USA

†Contributed equally to the work.


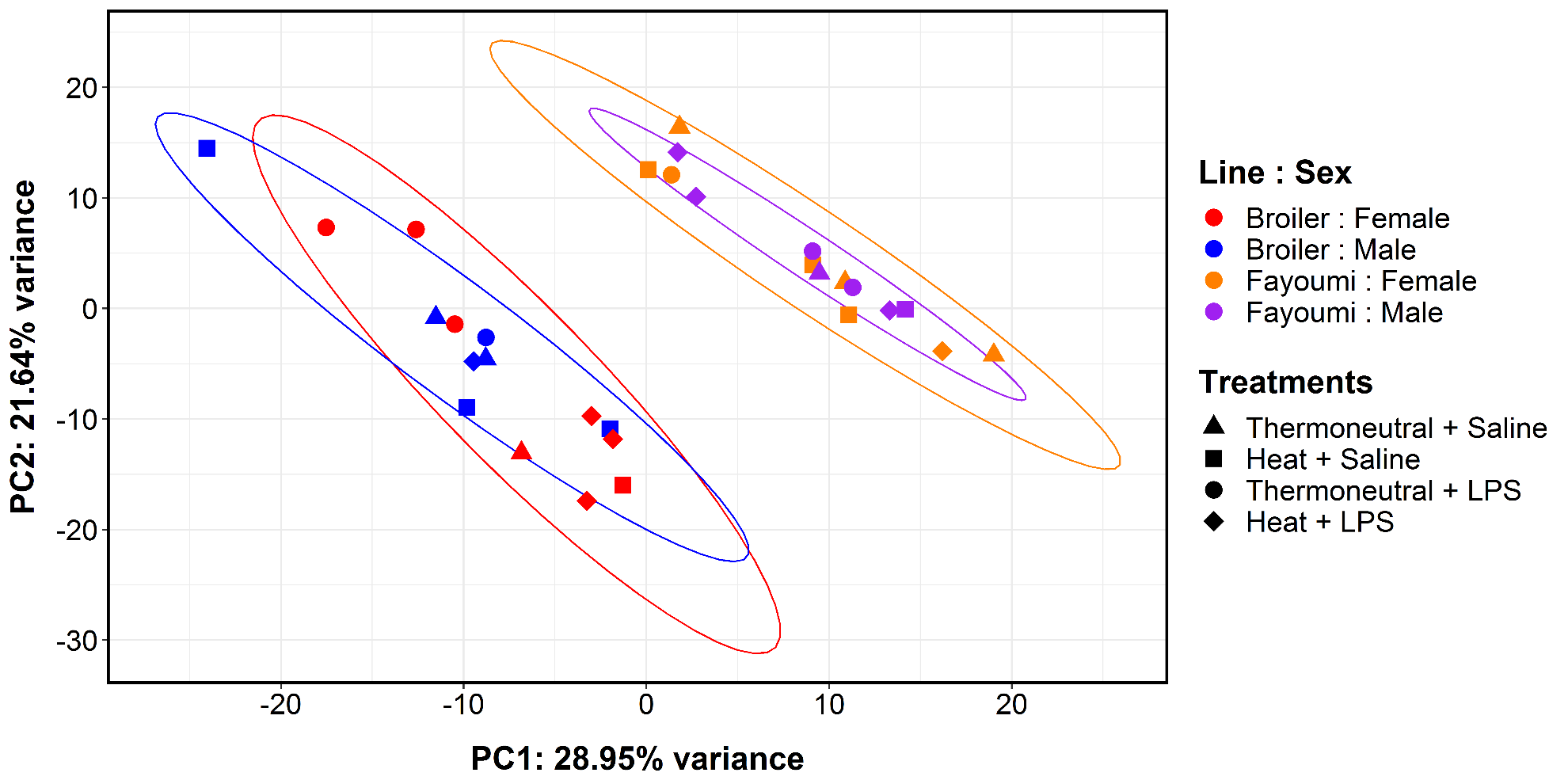


**Supplementary Figure S1.** Excluding genes on Z and W chromosomes removed the sex effect from PCA. Principal component analysis (PCA) was performed on variance stabilized normalized read counts from the 300 most variable genes on the autosomes. Samples are designated by line and sex (colour) and by treatment (shape). Ellipses represent a 95% confidence interval. Lipopolysaccharide (LPS), principal component 1 (PC1), principal component 2 (PC2).

**
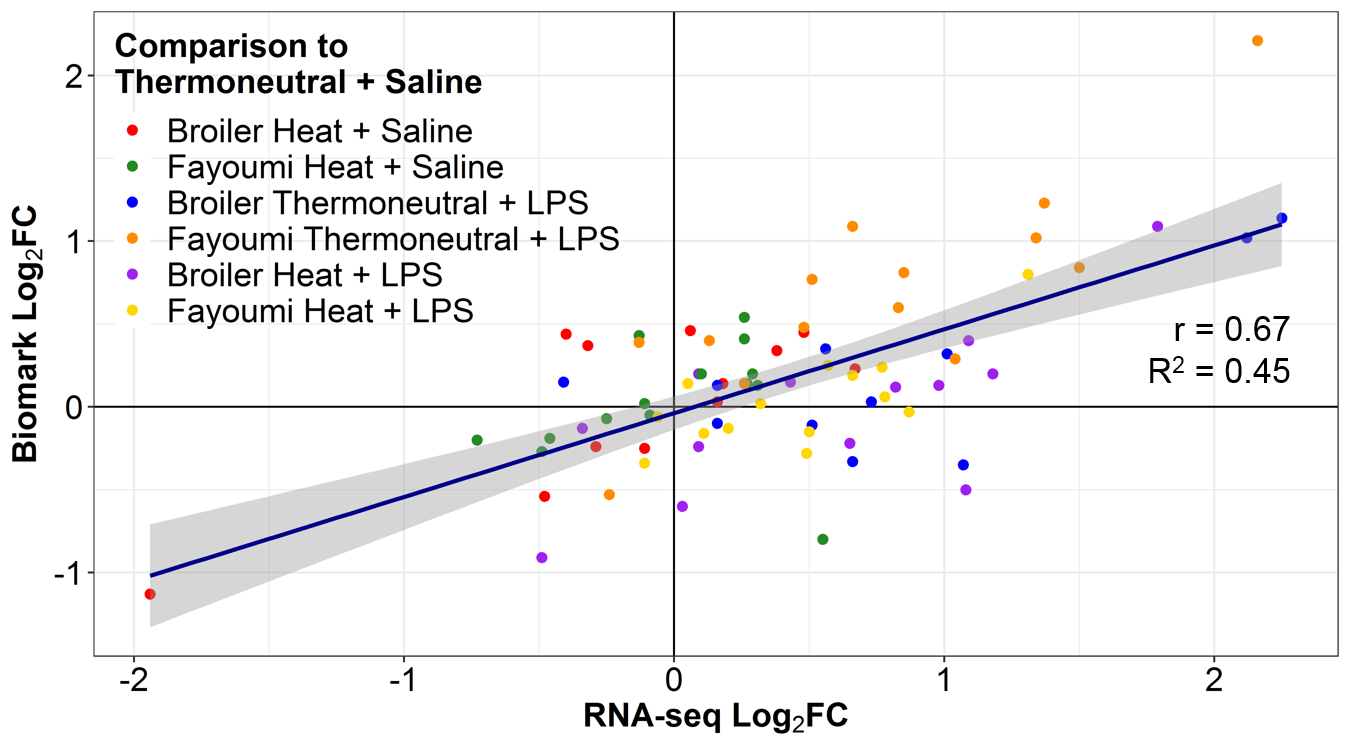
**

**Supplementary Figure S2.** Correlation between RNA-seq and Biomark qPCR in broiler for 14 genes. Log_2_FC from Biomark qPCR (2^-ΔΔ^*^C^*^t^ method) and RNA-seq (DESeq2) were compared by Pearson correlation. See Monson et al., 2018^29^ for the list of genes and primer sequences (excluded *IL10* and *IFNG* due to low expression in thymus). Quantitative PCR (qPCR), log_2_ fold change (log_2_FC), lipopolysaccharide (LPS).


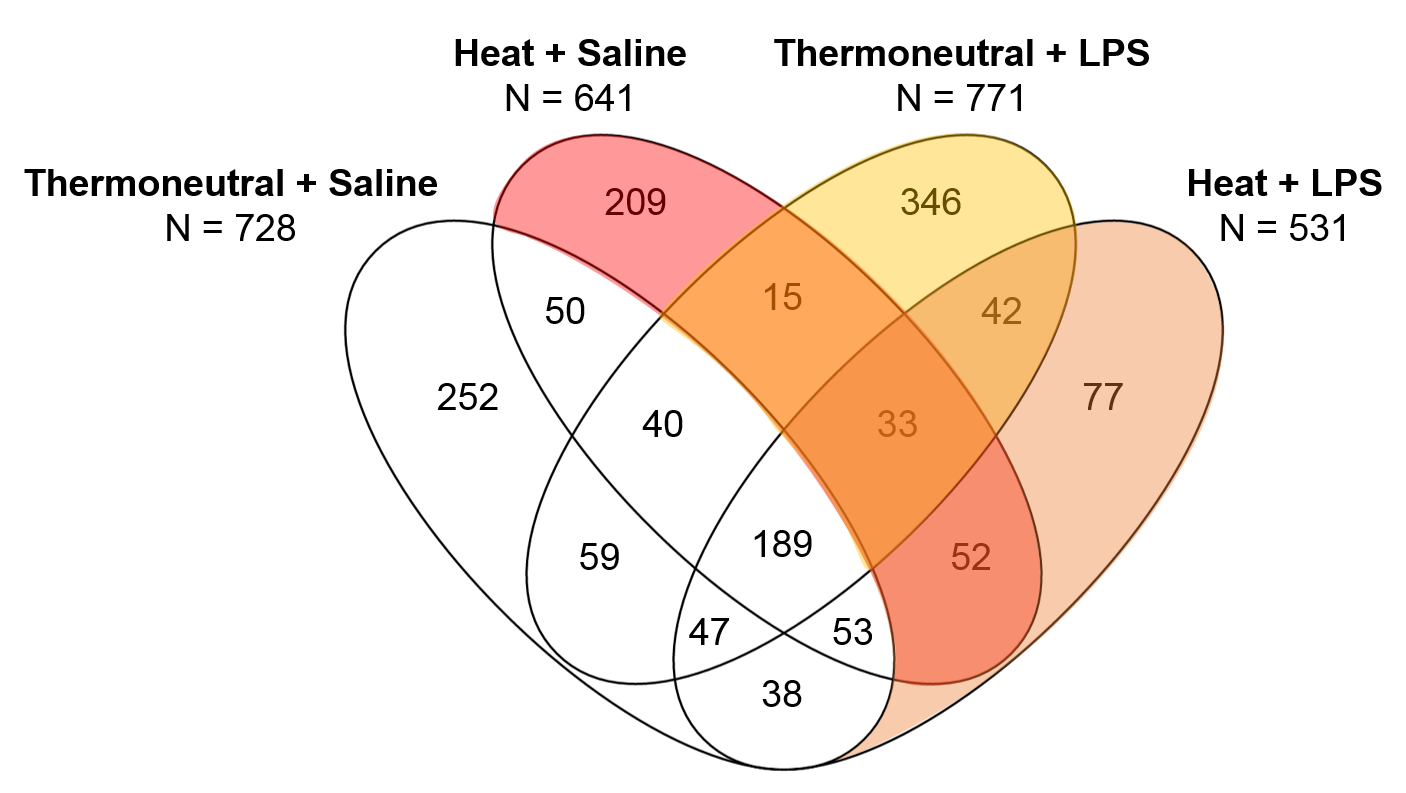


**Supplementary Figure S3.** Significant differential expression in broiler compared to Fayoumi overlapped between all treatments. The total number (N) and overlap in significant DE genes (q-value < 0.05, |log_2_FC| ≥ 1.0) are shown for each comparison between genetic lines. Differential expression (DE), lipopolysaccharide (LPS), log_2_ fold change (log_2_FC).

**Supplementary Dataset S1.** Results of RNA-seq, QC, and read mapping for each thymic dataset. (Excel file)

**Supplementary Dataset S2.** Significant differential expression in pairwise comparisons between treatments or chicken lines. (Excel file)

**Supplementary Dataset S3.** Overrepresented GO Biological Process terms in pairwise comparisons between treatments or chicken lines. (Excel file)

**Supplementary Dataset S4.** Activated or inhibited downstream functions predicted from pairwise comparisons between treatments or chicken lines. (Excel file)
